# Health-Related Quality of Life in Obese Presurgery Patients with and without Binge Eating Disorder, and Subdiagnostic Binge Eating Disorders

**DOI:** 10.1155/2013/878310

**Published:** 2013-03-13

**Authors:** Rita Marie Sandberg, Jens K. Dahl, Einar Vedul-Kjelsås, Bjørnar Engum, Bård Kulseng, Ronald Mårvik, Lasse Eriksen

**Affiliations:** ^1^Division of Psychiatry, Department of Nidaros DPS, St. Olavs University Hospital, 7440 Trondheim, Norway; ^2^Department of Neuroscience, Faculty of Medicine, NTNU, 7006 Trondheim, Norway; ^3^Division of Psychiatry, Department of Research and Development, St. Olavs University Hospital, Østmarka, 7006 Trondheim, Norway; ^4^Department of Psychology, Norwegian University of Science and Technology, 7491 Trondheim, Norway; ^5^Center of Obesity, St. Olavs University Hospital, 7006 Trondheim, Norway

## Abstract

*Objective*. To study health-related quality of life (HRQoL) in obese presurgery patients with binge eating disorder (BED) and with subdiagnostic binge eating disorder (SBED) compared to patients without eating disorders or SBED. *Method*. Participants were patients referred to St. Olavs University Hospital, Norway, for bariatric surgery. Eating Disorders in Obesity (EDO) questionnaire was used to diagnose BED and SBED. Short-Form Health Survey (SF-12) assessed health-related quality of life. Questionnaires were returned by 160 of 209 patients. The present study sample consisted of 143 patients (103 women and 40 men) as 17 patients did not complete the SF-12. *Results*. Patients with BED and patients with SBED both had significantly lower mental HRQoL, but not physical HRQoL, compared to patients without eating disorders. *Discussion*. The findings indicate that obese presurgery patients with BED, and also SBED, may have special treatment needs in regard to their mental health.

## 1. Introduction

The prevalence of obesity (defined with a BMI of ≥30) is increasing worldwide and is regarded as one of the biggest challenges for public health [1, 2]. Of the obese individuals who seek weight loss treatment, 16% to 30% report that they have an eating disorder [3], with the highest prevalence in patients seeking surgery treatment [3, 4]. Both obesity and eating disorders impair physical function and decrease subjective well-being [5, 6]. Based on this, obese individuals with a comorbid eating disorder may experience an extra burden. It is therefore of interest to study whether health-related quality of life (HRQoL) is different in obese presurgery patients with and without binge eating disorder (BED) or subdiagnostic binge eating disorders (SBEDs). 

### 1.1. HRQoL in Obesity and Eating Disorders

Impaired quality of life is often the reason obese individuals seek treatment [7]. The concept “HRQoL” is related to how the individual experiences his or her own health status [7, 8]. HRQoL is reported to be lower in obese individuals who seek treatment compared to the normal population [9] and obese individuals who do not seek treatment [10–12]. It may be a continuum, with lower HRQoL in the most demanding treatment as patients who seek surgery treatment report the lowest HRQoL score [12, 13], even when they are compared to patients with serious chronic somatic diseases [9]. 

For assessing HRQoL, the Medical Outcomes Study Short-Form Health Survey Scale SF-36 [14] and SF-12 [15] are well-validated and well-used generic instruments. In the present study SF-12 was applied. SF-12 is divided in two parts: one for mental health (MCS) and one for physical health (PCS). According to several studies, obesity causes a larger impairment in physical HRQoL compared to mental HRQoL (e.g., [5, 9]). Eating disorders, however, are found to impair mainly the mental HRQoL (e.g., [6, 16, 17]). The main difference between the obese patients with and without eating disorders has been observed as lower HRQoL in the obese patients with an eating disorder, and the impairment seems to be mostly in the mental domains of HRQoL (e.g., [4, 9]). However, Hsu et al. [18] found difference in physical HRQoL between patients with and without BED, but de Zwaan et al. [9] did not.

### 1.2. Eating Disorders in Obese Presurgery Patients

Binge eating disorder (BED) is prevalent in obese patients who seek surgery treatment [19]. However, a focus on BED alone will not contribute to a better understanding of how eating behavior affects weight-loss treatment [20] because bulimia nervosa (BN), eating disorders not otherwise specified (EDNOS), and subdiagnostic binge eating disorders (BED minus one criterion = SBED) have also been reported in many of the obese presurgery patients [18, 19, 21]. Considering the high prevalence of binge eating [19], excluding patients who do not fulfill all criteria for BED would clearly exclude a large number of obese patients with disordered eating. Inclusion of patients who do not fulfill all criteria for an eating disorder would provide more information concerning subdiagnostic eating disorders and clinical impairment following this condition [20]. The use of the Oxford criteria for diagnosing BED (one binge eating episode per week on average during the last six months) has been suggested [22]. Some studies have used this criterion and thus, in a way, included SBED within BED. Results from a community study regarding subthreshold binge eating disorders indicated that patients with one binge eating episode per week may show the same impairment as patients with two binge eating episodes [23]. It has also been shown that obese patients with SBED are as vulnerable as patients with EDNOS or BED in anxiety and neuroticism [24]. We found only three studies that included the full range of eating disorders in obese individuals. One study investigated obese patients seeking nonsurgical weight-loss treatment [20], and the other two studies investigated obese patients seeking surgery treatment [21, 24].

The aim of the present study is to investigate whether obese presurgery patients categorized with BED or with SBED have different mental and physical HRQoL in SF-12 (MCS and PCS) compared to obese presurgery patients without eating disorders or SBED. 

### 1.3. The Directive Hypothesis

Patients categorized with BED or with SBED will show significantly lower mental quality health-related quality of life than those who have no ED and no SBED.

### 1.4. The Nondirective Hypothesis

Patients categorized with BED or with SBED will show significantly different physical quality health-related quality of life than those who have no ED and no SBED.

All relevant eating disorders in obese presurgery patients were included in the explorative part of the study, where variations in gender, civil status, onset of obesity, BMI, age and education were investigated in regard to scores on SF-12.

## 2. Method

### 2.1. Participants, Design, and Procedure

The participants were patients referred to St. Olavs University Hospital for laparoscopic gastric bypass surgery. All patients in the present study fulfilled the inclusion criteria for surgery: BMI between 35 and 40 with one obesity-associated medical comorbidity or a BMI ≥40. Exclusion criteria were pregnancy, substance abuse, severe mental disorders (except eating disorders), prior bariatric surgery, and dependence on wheelchair or crutches. Participation in the study did not affect the preoperative screening and approval process.

The Norwegian Regional Ethics Committee (REK) approved the study, which has a cross-sectional design with one assessment time. A total of 209 patients who were eligible for the study gave their informed consent to participate. The patients were mailed self-report questionnaires. One reminder was given. A total of 160 questionnaires were returned, giving a 76.5% response rate. 

### 2.2. Diagnostic Classification of Eating Disorders

Eating disorder diagnoses were identified using the questionnaire Eating Disorders in Obesity (EDO). EDO consists of 11 questions that measure eating disorders based on DSM-IV criteria. No questions concerning underweight were included as EDO is used to assess eating disorder symptoms in obese patients [25]. The participants filled in the questionnaire at home and posted it. The term “binge eating” is vague, and because it can be interpreted in various ways, the EDO provides a definition based on DSM-IV criteria before the respondents complete the questionnaire. This may reduce false positive cases [25]. EDO has shown good criterion validity compared to the Eating Disorder Examination (EDE) interview [25], which is considered the “gold standard” of eating disorders measurement [26]. EDO has the ability to detect possible clinical cases concerning eating disorders as well as binge eating [25].

Three diagnostic categories of eating disorders were found: BN, BED, and EDNOS. Patients meeting DSM-IV criteria for BN, BED, or EDNOS criteria were included in ED, the eating disorder group. The patients who were categorized with EDNOS were one criterion short of full BN diagnosis and resemble atypical bulimia nervosa in ICD-10 [27]. Patients who lacked one criterion from the DSM-IV binge eating disorder were included in the subdiagnostic binge eating disorder (SBED) group. There were three ways of missing one criteria for the BED: (1) reduced frequency regarding binge eating episodes (less than 2 days a week for 6 months), (2) binge eating only associated with only two of the following: eating more rapidly, eating until feeling uncomfortably full, eating large amounts of food when not physically hungry, eating alone and feeling depressed or guilty after eating, or (3) reporting not feeling depressed or guilty (excessive) after overeating. Accordingly, there were three ways to be categorised with SBED. Patients who reported no BED or no subdiagnostic binge eating disorder were included in the no ED or SBED group. In the original sample of 160 patients, 157 could be classified with or without an ED or SBED. Three patients could not be diagnosed because of missing responses on EDO. They were therefore included in the no-ED group. Twenty-four of the patients were classified as having an ED (16.8%), and by adding SBED, the group consisted of 44 patients (30.8%). Of these, one patient was classified with BN (0.7%), five patients with EDNOS (3.5%), 18 with BED (12.6%), and 20 with SBED (14%). A total of 99 patients were classified as having no ED or SBED. 

### 2.3. Comparison Groups

**Figure 1:**
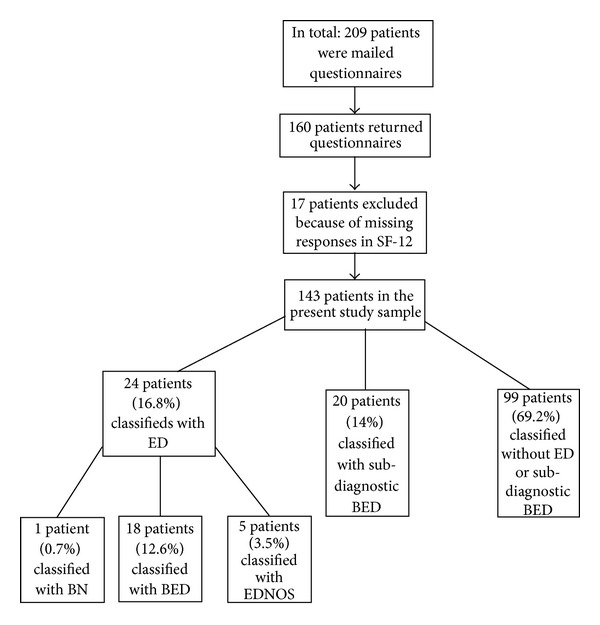
Flowchart for subject recruitment and eating disorder diagnosis in the present study.

The main comparison of the present study is between the categories BED and SBED and those without ED or SBED. Because few patients fulfilled the criteria for BN and EDNOS, these categories were excluded. In the explorative part of the study, all categories are included to give a more comprehensive picture of the EDs among patients seeking bariatric surgery. See Figure 1.

The characteristics for the BED group, the SBED group, and the no ED or SBED group are presented in Table 1.

### 2.4. Assessment

HRQoL was measured using the Medical Outcomes Study 12-item Short-Form Health Survey (SF-12). SF-12 is a short version of the well-validated generic self-report questionnaire SF-36. SF-12 consists of 12 of the 36 items from SF-36 [15] distributed on eight domains assessing activity and role limitations due to physical or emotional problems. The mental component summary (MCS) has six items and the physical component summary (PCS) has the same. The higher the score, the greater the quality of life. Use of the summary measures (MCS and PCS) instead of a profile with the eight domains is recommended [15, 28]. In the US population, the SF-12 can explain 90% of the variance in the PCS and MCS summary measures from SF-36 [15]. SF-12 summary measures also correlated highly with SF-36 measures when tested in nine European countries, including Norway [28]. Even though a reduction in precision is seen when only 12 items are included in the domains that measure health, it is considered a good measure to assess overall physical and mental health [15, 28]. Good reliability and validity in SF-12 have been documented by Ware et al. [15]. Test-retest reliability for PCS is reported to be 0.89 in the USA and 0.86 in the UK. For the MCS the test-retest reliability is reported to be 0.76 in the USA and 0.77 in the UK [15]. Gandek et al. [28], using data from general population surveys (*n* = 1483 to 9151) in nine European countries, reported product-moment correlation between SF-36 summary measures and SF-12 summary measures (standard- and country-specific) to be very high, ranging from 0.94 to 0.97 (MCS) and 0.94 to 0.96 (PCS) [28]. Because of missing responses on the SF-12, 17 patients were excluded [29], which left 143 patients in the present study sample. 

### 2.5. Statistical Analysis

A one-way ANOVA between groups was applied to explore the influence of eating disorders symptoms on health-related quality of life, as measured by the SF-12 (MCS and PCS). Obese presurgery patients were split in three groups (BED, SBED, and no ED or SBED). Post hoc comparisons with Bonferronis test were applied. Bonferronis test was chosen since it controls the type 1 error rate very well and has more power when applied for few groups.

The statistical tests are two-tailed in spite of the directional hypotheses, as there is a close link between the use of a confidence interval and a two-sided significance test [30]. The effect size of eta-squared was calculated and Cohen's norms were applied [31]. A 95% confidence interval was computed and interpreted. SPSS 18.0 for MAC was applied when data were calculated. 

## 3. Results

In a one-way between-groups analysis of variance there was a statistically significant difference at the *P* < .05 level in MCS scores for the three groups (*F* (2,134) = 6.50,  *P* = .002). The effect size, calculated using eta-squared, *η* ^2^ = .09.

Post hoc comparisons using the Bonferroni test indicated that the mean MCS score was 8.36 points lower (95% CI −16.01, −.714; *P* = .027) for the BED group as compared with the group with no ED or SBED. Mean MCS score was 8.57 points lower (95% CI −15.88, −1.25; *P* = .016) for the SBED group compared to the no ED or SBED. Mean MCS score was  .21 points lower (95% CI −9.49, 9.90; *P* = 1.00) for the BED group compared to the group with SBED.

There was a nonsignificant difference at the *P* < .05 level in PCS scores for the three groups (*F* (2,134) = .61,  *P* = .54,  *η* ^2^ = .009). 

In the explorative part of the study, no difference in MCS or PCS scores in SF-12 was found in regard to gender, civil status, onset of obesity or high or low BMI or age (median split) by independent *t*-test (two-tailed). Patients with high education (one year or more at college/university) had significantly higher scores in the physical scale in the SF-12 (PCS) (*M* = 36.75, SD = 12.15) than patients with low education (*M* = 32.11, SD = 10.57, *t* (141) = 2.20,  *P* < .030,  *d* = 0.4, mean difference: 4.64, 95% CI 0.46, 8.83). No significant difference was found in relation to mental health-related quality of life (MCS).

## 4. Discussion

The results show that the directive hypothesis was supported: lower scores in mental health were found among the patients with BED and with SBED, on the SF-12, compared to the no ED or SBED group. The nondirective hypothesis about physical health, however, was not supported. In the exploratory part of the study, the patients with high education indicated significantly higher scores than patients with low education in the physical HRQoL. There were no significant differences indicated in the two parts of SF-12 in regard to gender, onset of obesity, living with or without a partner, age, or BMI (median split). 

### 4.1. HRQoL in Obese Patients with Clinical and Subdiagnostic Binge Eating Disorder

Prior studies have found lower HRQoL in obese presurgery patients with BED (e.g., [9, 18]) compared to the obese presurgery patients without BED. Results in the present study support previous findings. In addition results indicate a need to include subdiagnostic binge eating disorder (SBED) in the psychological assessment prior to surgery, as these patients report equally reduced mental health-related quality of life as patients with BED. This suggests that the patients who lack one criterion for BED have an impaired mental HRQoL probably much like those with full criteria for BED. The finding that SBED appears to be quite similar to full criteria of BED is in line with the finding from other studies (e.g., [23, 32]). The results of the present study and other studies [6, 20] signal that the presence of symptoms of an eating disorder might impair quality of life. Identifying patients with SBED is relevant as they may have lower mental HRQoL than patients without eating disorders and have impairments that are of clinical importance [27].

### 4.2. Eating Disorders, Psychiatric Comorbidity and HRQoL in Obese Patients

While obesity may cause impaired physical health [9, 33], having eating disorders may cause an extra burden that impairs mental HRQoL [9]. In addition to eating disorders, however, a high occurrence of comorbid Axis I disorders, like anxiety and depression, has been found in obese presurgery patients (e.g., [34]). This type of comorbidity may also contribute to low mental HRQoL [6, 9]. Therefore, it may be difficult to determine whether low HRQoL is caused by eating disorders or other psychiatric disorders [9, 17]. 

Hsu et al. [18] found differences regarding physical and mental HRQoL between patients with extreme obesity (BMI > 40) with BED versus patients with no BED. The impairment seemed not to be associated with differences in gender, age, or BMI. The findings in the present study support the results in the Hsu et al. study [18]: BMI, obesity onset, and demographic variables cannot explain why the presurgery patients with eating disorders have a lower mental HRQoL than the patients without. However, de Zwaan et al. [9] found no differences between pre-surgical obese patients with BED and those with no BED in impaired mental HRQoL when depression and (low) self-esteem were controlled for. Apparently, the results are inconclusive regarding how various eating disorder diagnoses and general psychopathology affect health-related quality of life [6]. 

### 4.3. Binge Eating Disorder and Subdiagnostic Binge Eating Disorder and Physical HRQoL (PCS)

Finding no differences in physical HRQoL (PCS) between the obese patients with BED or with SBED versus those without ED or SBED is in accordance with the finding of a study that investigated HRQoL and BED [9], but not with the Hsu et al. [18] study. Obesity per se seems to have a profound and direct effect on physical HRQoL, and impairment in physical HRQoL has been reported to increase with an increasing BMI [33].

### 4.4. Explorative Study

Obese patients with low education (no college or university) indicated poorer physical HRQoL (PCS) than obese patients with higher education. This is most probably a random association, since it was the only significance among 12 tests in the explorative part of the present study and thus only represents a possible hypothesis in a confirmatory study in the future.

More than 70% of the patients in the present sample are in the low education group. This seems to be a high proportion, considering that de Zwaan et al. [35] reported that a total of 75.9% of their pre-surgical patients had some college education and 52.8% had completed a college degree. 

### 4.5. Limitations and Strengths

The response rate of 68.4% in the present study is a limitation. Nevertheless, a response rate of 68.4% by mail is regarded as good according to Berger [36] and Judd et al. [37]. 

A possible limitation of the study is that diagnosis of eating disorders is based on a self-report instrument (EDO). Using interviews is reported to produce lower estimates for ED than using questionnaire-based evaluations [38, 39]. An interview like the Eating Disorder Examination is also preferred as an interviewer could have helped the patients to correctly understand the questions, definitions, and/or criteria [39]. The drawbacks with interview-based diagnosis are the considerable time and financial resources required to complete them. de Zwaan et al. [38] reported a diagnostic agreement with regard to the absence or presence of BED that represented a fair-to-good agreement beyond chance in a sample of 100 obese women. The expert rating with an interview identified 43 cases of BED, while 40 cases were identified with a self-report questionnaire.

A strength of anonymous self-report is that it may reduce patients resistance to provide correct information concerning eating behavior [40, 41]. In addition, participating in the present study did not affect the preoperative screening or approval process for the surgery, so the risk for underreporting binge eating or eating disorders may therefore be reduced. 

The use of self-reported weight and height may be questioned. It has been suggested that individuals who are overweight and severely overweight have a tendency to underreport weight and overreport height, which could lead to an inaccurate estimate of obesity [42, 43]. Despite this, self-reported measures have been found to be adequate [44]. 

An additional limitation may be the use of only a generic measure, the SF-12, as it provides a general assessment concerning physical and mental HRQoL. A generic measure may be less sensitive than a condition-specific measurement [6]. The HRQoL as a concept is not disease-specific, and a generic measurement makes possible the comparison of results with various kinds of groups or disorders. SF-12 has proven to be a good measurement of HRQoL [15, 28]. It has the advantage of a short completion time (2-3 minutes) and is therefore a low burden on the respondent and, in addition, provides a substantial amount of information [15]. 

The study does not control for possible confounding variables in assessing group differences in SF-12. This represents a limitation.

The cross-sectional study design prevents any cause-effect conclusions. Other research designs are needed to increase the cause-effect knowledge between eating disorders and mental and physical health-related quality of life (HRQoL) in obese presurgery patients. 

## 5. Conclusions

Results from the present study show that obese presurgery patients with binge eating disorder (BED) and with subdiagnostic binge eating disorders (SBED) both report significantly lower mental health-related quality of life (HRQoL), compared to obese presurgery patients with no eating disorders. Former studies have indicated that an eating disorder may have a negative influence on mental HRQoL in patients waiting for bariatric surgery. The present study highlights that patients with subdiagnostic binge eating disorder may, like the patients with binge eating disorders, need additional treatment interventions that focus on their mental health to improve their mental HRQoL.

## Figures and Tables

**Table 1:** Characteristics for the binge eating disorders group, the sub-diagnostic binge eating disorders group, and the noneating disorders group.

	Binge eating disorders	Sub-diagnostic BED	Noneating disorders
	*n* = 18	*n* = 20	*n* = 99
	Mean (SD)	Mean (SD)	Mean (SD)
Age	39.83 (8.38)	35.45 (10.11)	42.14 (10.15)
BMI	47.21 (6.22)	48.25 (6.17)	47.82 (5.91)
MCS^1^	36.56 (15.06)	36.36 (13.16)	44.92 (11.59)
PCS^2^	35.44 (12.79)	34.77 (11.14)	32.79 (10.68)

	*n* (%)	*n* (%)	*n* (%)

Obesity onset in childhood	12 (68%)	16 (80%)	67 (67.7%)
Living with partner	13 (72.2%)	15 (75%)	61 (61.6%)
High education^3^	2 (11%)	8 (40%)	24 (24.2%)

^1^Mental component summary.

^2^Physical component summary.

^3^1 year or more at college/university.
